# Effects of intraoperative PEEP optimization on postoperative pulmonary complications and the inflammatory response: study protocol for a randomized controlled trial

**DOI:** 10.1186/s13063-017-2116-z

**Published:** 2017-08-11

**Authors:** Zoltán Ruszkai, Erika Kiss, Ildikó László, Fanni Gyura, Erika Surány, Péter Töhötöm Bartha, Gergely Péter Bokrétás, Edit Rácz, István Buzogány, Zoltán Bajory, Erzsébet Hajdú, Zsolt Molnár

**Affiliations:** 1 Department of Anaesthesiology and Intensive Care, Péterfy Sándor Hospital, Péterfy Sándor u. 8-20, Budapest, 1076 Hungary; 2 Department of Anaesthesiology and Intensive Therapy, University of Szeged, Semmelweis u. 6, Szeged, 6725 Hungary; 3 Department of Urology, Péterfy Sándor Hospital, Péterfy Sándor u. 8-20, Budapest, 1076 Hungary; 4 Department of Urology, University of Szeged, Kálvária sgt.57, Szeged, 6725 Hungary

**Keywords:** Positive end-expiratory pressure, Static pulmonary compliance, Lung-protective ventilation, Radical cystectomy, Postoperative pulmonary complications, Procalcitonin

## Abstract

**Background:**

Patients undergoing general anesthesia and mechanical ventilation during major abdominal surgery commonly develop pulmonary atelectasis and/or hyperdistention of the lungs. Recent studies show benefits of lung-protective mechanical ventilation with the use of low tidal volumes, a moderate level of positive end-expiratory pressure (PEEP) and regular alveolar recruitment maneuvers during general anesthesia, even in patients with healthy lungs. The purpose of this clinical trial is to evaluate the effects of intraoperative lung-protective mechanical ventilation, using individualized PEEP values, on postoperative pulmonary complications and the inflammatory response.

**Methods/design:**

A total number of 40 patients with bladder cancer undergoing open radical cystectomy and urinary diversion (ileal conduit or orthotopic bladder substitute) will be enrolled and randomized into a study (SG) and a control group (CG). Standard lung-protective ventilation with a PEEP of 6 cmH_2_O will be applied in the CG and an optimal PEEP value determined during a static pulmonary compliance (Cstat)-directed PEEP titration procedure will be used in the SG. Low tidal volumes (6 mL/Kg ideal bodyweight) and a fraction of inspired oxygen of 0.5 will be applied in both groups. After surgery both groups will receive standard postoperative management. Primary endpoints are postoperative pulmonary complications and serum procalcitonin kinetics during and after surgery until the third postoperative day. Secondary and tertiary endpoints will be: organ dysfunction as monitored by the Sequential Organ Failure Assessment Score, in-hospital stay, 28-day and in-hospital mortality.

**Discussion:**

This trial will assess the possible benefits or disadvantages of an individualized lung-protective mechanical ventilation strategy during open radical cystectomy and urinary diversion regarding postoperative pulmonary complications and the inflammatory response.

**Trial registration:**

ClinicalTrials.gov, ID: NCT02931409. Registered on 5 October 2016.

**Electronic supplementary material:**

The online version of this article (doi:10.1186/s13063-017-2116-z) contains supplementary material, which is available to authorized users.

## Background

Patients undergoing general anesthesia and mechanical ventilation during major abdominal surgery commonly develop pulmonary atelectasis and/or hyperinflation of the lungs leading to complications either intraoperatively or in the postoperative period, resulting in ventilator-induced lung injury (VILI) [1, 2].

Lung-protective mechanical ventilation (LPV), by applying “low” tidal volumes (TV = 6 mL/Kg of ideal bodyweight, IBW), optimal positive end-expiratory pressure (PEEP) and regular alveolar recruitment maneuvers (ARM) in case of acute respiratory distress syndrome (ARDS) have been shown to be advantageous in critically ill patients. Recent studies have also shown positive results of LPV and regular ARM during general anesthesia in patients with healthy lungs [3, 4]. The main advantages of this strategy are improved gas exchange and prevention of either pulmonary atelectasis or VILI [5–7]. However, the effects of applying an optimal level of PEEP have not entirely been evaluated.

There are several types of PEEP titration methods such as dead space fraction (Vds/Vt)-guided or static pulmonary compliance (Cstat)-directed techniques [8–12].

Theoretically, in patients with healthy lungs, during general anesthesia and mechanical ventilation, inadequate PEEP values may lead to decreased pulmonary compliance and gas exchange disorders due to pulmonary atelectasis and/or hyperinflation of the lungs. In our clinical trial, optimal PEEP values will be determined during a static pulmonary compliance-directed PEEP titration procedure to protect from hyperdistention, and regular ARMs will be performed using the sustained airway pressure by the continuous positive airway pressure (CPAP) method, applying 30 cmH_2_O of PEEP for 30 s, to prevent atelectasis [5, 13, 14].

On the one hand major abdominal surgery induces an inflammatory response that is necessary for postoperative recovery (e.g., wound healing), but on the other hand an overwhelming inflammatory response may also lead to adverse events (AEs) such as organ dysfunction [15–19]. Radical cystectomy is considered major surgery; hence, there is an increased risk of postoperative complications. Inappropriate mechanical ventilation during general anesthesia can also lead to an amplified inflammatory response, which theoretically may worsen the postoperative outcome via several mechanisms. However, the relationship between LPV and the postoperative inflammatory response after radical cystectomy has not been investigated yet.

There is strong correlation between the degree of inflammatory response and serum procalcitonin (PCT) concentrations [20, 21]; hence, there is some rationale in the belief that monitoring the inflammatory response by regular PCT measurements in the postoperative period reflects the host response. Therefore, there is some rationale in monitoring PCT kinetics as an indicator of the host inflammatory response.

The aim of this investigator-initiated, double-center, single-blinded (subject), prospective, randomized controlled trial is to evaluate the effects of intraoperative LPV, applying an individually titrated optimal PEEP, on postoperative pulmonary complications (PPC) and the inflammatory response in patients undergoing radical cystectomy and urinary diversion (ileal conduit or orthotopic bladder substitute). We hypothesized that optimizing intraoperative mechanical ventilation (incorporating LPV, ideal PEEP and ARM) can attenuate the inflammatory response as compared to conventional modes of mechanical ventilation, and hence may result in improved postoperative oxygenation, prevent the occurrence of VILI, and reduce the incidence of organ dysfunction. These anticipated advantages may also improve postoperative recovery and survival rates, shorten in-hospital stay and reduce health care-related costs.

## Methods/design

### Objectives of the study

The main objectives of this trial are to compare the effects of a standard LPV applying 6 cmH_2_O of PEEP to a LPV using an individually titrated optimal PEEP on: (1) oxygenation and PPC, (2) the degree of inflammatory response evaluated by early PCT kinetics (0, 2, 6, 12, 24, 48 and 72 h after surgical incision) and (3) to evaluate the relationship between the degree of inflammation and postoperative pulmonary and extrapulmonary complications.

### Study endpoints

The primary outcome variables are PPC and PCT kinetics. PPC are defined as new infiltrates or atelectasis on a chest X-ray, abnormal breathing sounds on auscultation, respiratory failure defined as PaO_2_/FiO_2_ < 300 or the need for noninvasive or invasive ventilatory support within the first three postoperative days. PCT kinetics will be evaluated during and after surgery. Blood samples will be taken at 0, 2, 6, 12, 24, 48 and 72 h after surgical incision. According to recent data it is expected that PCT values will peak at approximately 24 h after surgery and that they should decline by approximately 50% daily in the case of an uneventful postoperative course. Therefore, in addition to the absolute values the change between T_0_–T_24_–T_48_ will also be evaluated [16, 22].

Secondary outcome variables are extrapulmonary complications: incidence of circulatory failure, gastrointestinal and renal dysfunction, hematologic and coagulation disorders and infection (Table 1).

**Table 1:** Secondary endpoints

Endpoint	Time frame	Detailed description
Circulatory failure	28 days	Hypotension – MAP < 65 mmHg
		Severe cardiac arrhythmia – 40/min < HR > 150/min
		ScvO_2_ < 70%
		dCO_2_ > 7 mmHg
		Serum lactate > 2 mmol/L
		Severe metabolic acidosis (actual bicarbonate < 18 mmol/L)
		Acute coronary syndrome
		Acute left ventricular failure
		Pulmonary embolism
		Cardiac arrest
Gastrointestinal dysfunction	28 days	Constipation
		Ileus
		Anastomotic leakage
		Reoperation
		Disorders of liver function
Renal dysfunction	28 days	RIFLE criteria
Hematologic and coagulation disorders	28 days	Severe bleeding
		Coagulopathy – INR > 1.5
Infection	28 days	Any infection except from pneumonia

*MAP* mean arterial pressure, *HR* heart rate, *ScvO*_*2*_ central venous oxygen saturation, *dCO*_*2*_ central venous-to-arterial carbon dioxide gap, *INR* International Normalized Ratio

Tertiary endpoints are intensive care unit (ICU) days, in-hospital stay, in-hospital and 28-day mortality.

### Study design

**Fig. 1:**
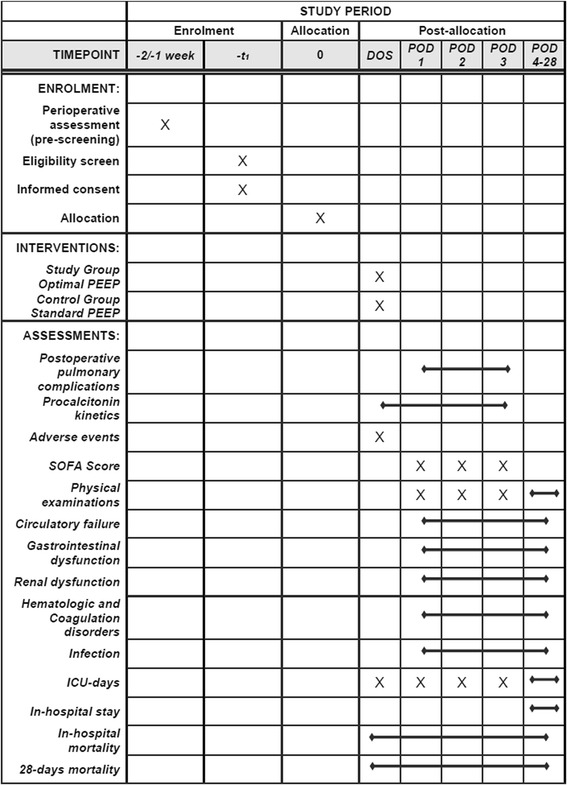
Standard Protocol Items: Recommendation for Interventional Trials (SPIRIT) schedule of enrollment, interventions and assessments. *DOS* day of surgery, *POD* postoperative day, *SOFA* Sequential Organ Failure Assessment, *ICU i*ntensive care unit

This is an investigator-initiated, double-center, parallel-group, single-blinded, interventional, prospective, randomized controlled trial conducted at the Department of Anesthesiology and Intensive Care of Péterfy Sándor Hospital Budapest and at the Department of Anesthesiology and Intensive Therapy of University of Szeged. The first patient will be randomized in October 2016. This protocol conforms to the Consolidated Standards of Reporting Trials (CONSORT) guidelines. Figure 1 shows the Standard Protocol Items: Recommendation for Interventional Trials (SPIRIT) schedule of enrollment, interventions and assessments. The SPIRIT 2013 Checklist is given in Additional file 1.

### Blinding, data collection, randomization and record-keeping

This is a single-blinded (participant) study. Patient data, intraoperative and postoperative measurements, fluid balance, respiratory parameters, laboratory results and clinical status (Sequential Organ Failure Assessment (SOFA) score) will be collected onto Case Report Forms (CRF). CRF and the patient evaluation chart will not be assessed in front of the patient.

Participants will be randomized to the SG or CG in a ratio of 1:1. Randomization will be carried out by a computer-generated blocked randomization list with 10 blocks of four patients per block. Allocation will be stored in sealed, opaque and numbered envelopes. Participants will be included and allocated in numerical order.

All original records (CRF and relevant correspondence) will be archived and secured for 15 years, and then destroyed according to the hospital standards concerning destruction of confidential information.

### Selection of the participants

Patients with bladder cancer scheduled for open radical cystectomy and urinary diversion will be screened and recruited during routine perioperative assessment. Participants fulfilling the inclusion criteria will be asked for their signed informed consent. Withdrawal of consent may be initiated by the participant at any time during the trial.

Inclusion criteria are age over 18 years, patients with bladder cancer undergoing radical cystectomy and urinary diversion (ileal conduit or orthotopic bladder substitute) and provision of signed informed consent.

Exclusion criteria are age below 18 years, American Society of Anesthesiologists (ASA) physical status IV, history of severe chronic obstructive pulmonary disease (COPD, GOLD grades III or IV), history of severe or uncontrolled bronchial asthma, history of severe restrictive pulmonary disease, pulmonary metastases, history of any thoracic surgery, need for thoracic drainage before surgery, renal replacement therapy prior to surgery, congestive heart failure (NYHA grades III or IV), extreme obesity (Body Mass Index, BMI > 35 Kg/m^2^) and lack of patient’s consent.

### Time course of the study

#### Preoperative assessment and admission

During standard institutional preoperative assessment, the patient’s eligibility for radical cystectomy and urinary diversion will be evaluated. Medical history, laboratory and chest X-ray or computed tomography (CT) scan, 12-lead electrocardiogram (ECG), ASA physical status, BMI, Respiratory Failure Risk Index (RFRI), nutritional risk screening (NRS 2002 tool) and, if required (in case of history of smoking or coronary artery disease), results of spirometry, echocardiography and ergometry will be recorded. Participants fulfilling the inclusion criteria will be asked for their signed informed consent.

After admission to the Department of Urology (on the day before surgery) a central venous catheter will be placed, a blood sample will be taken from included patients for baseline levels of PCT (T_0_), a chest X-ray will be performed and, if there are no exclusion criteria, patients will be randomized into one of the study groups. Patients will be given oral carbohydrate loading (maltodextrin) 12, 8 and 2 h before surgery, 1000 mL of crystalloid solution will be given and antimicrobial prophylaxis will be introduced using ciprofloxacin and metronidazole 30 min before surgical incision. Antimicrobial prophylaxis will be continued for 72 h (2 × 400 mg ciprofloxacin and 3 × 500 mg metronidazole per day). Deep vein thrombosis prophylaxis will be carried out using low-molecular-weight heparin (LMWH).

#### Intraoperative care

Before induction of anesthesia an epidural catheter and an arterial cannula will be inserted for invasive arterial blood pressure monitoring and blood gas sampling.

Immediately after induction of anesthesia and orotracheal intubation, once a steady state has been reached (Table 2), all patients will be submitted to an ARM using the sustained airway pressure by the CPAP method, applying 30 cmH_2_O PEEP for 30 s. After ARM, PEEP will be set to 6 cmH_2_O in the CG (“standard PEEP”) and LPV (TV = 6 mL/Kg IBW, FiO_2_ = 0.5) will be performed. In the SG (“optimal PEEP”) PEEP will be determined during a Cstat-directed decremental PEEP titration procedure. During surgery ARM will be repeated and arterial and central venous blood gas samples (ABGs, CVBGs) will be evaluated every 60 min. In case of decreased oxygen saturation (SpO_2_ < 94%) rescue ARM will be performed using a FiO_2_ of 1.0. PCT levels will be measured 2, 6, 12, 24, 48 and 72 h after surgical incision.

**Table 2:** Steady state after induction of anesthesia

	Parameter	Value
Hemodynamics	Mean arterial pressure	65 mmHg < MAP < 90 mmHg
	Heart rate	50/min < HR < 100/min
Ventilation	SpO_2_	≥96%
	EtCO_2_	35–40 mmHg
Anesthetics	EtSevo	1.0 MAC

*MAP* mean arterial pressure, *HR* heart rate, *SpO*_*2*_ peripheral capillary oxygen saturation, *EtCO*_*2*_ end-tidal carbon dioxide partial pressure, *EtSevo* end-tidal sevoflurane concentration, *MAC* minimal alveolar concentration

Arterial blood pressure, heart rate (HR) and end-tidal carbon dioxide tension (EtCO_2_) will be monitored continuously. Cstat, airway resistance (Raw), Vds/Vt, core temperature and train-of-four relaxometry data will be recorded every 15 min.

During surgery, in cases of hypotension, intravenous norepinephrine will be started to maintain mean arterial pressure above 65 mmHg. For intraoperative fluid management patients will receive 3 mL/Kg/h of balanced crystalloid solution until end of surgery. In cases of bleeding, a 200-mL colloid (hydroxyethyl starch, HES) solution bolus and crystalloid substitution will be given. Packed red blood cell (PRBC) transfusion will be given whenever the attending anesthetist feels it necessary.

#### Postoperative care

After extubation, patients will be admitted to the ICU. ABGs and CVBGs will be collected and evaluated (pH, base excess (BE), standard bicarbonate (stHCO^3−^), ScvO_2_), PaO_2_/FiO_2_ and central venous-to-arterial carbon dioxide gap (dCO_2_) will be calculated every 6 h until 72 h after surgery. On the first postoperative day (POD), a chest X-ray will be performed and repeated on the following days if the development of pulmonary complications are suspected. The chest X-ray will be evaluated by an independent, trained radiologist who will not be involved in the study. Continuous epidural analgesia and intermittent intravenously administered analgesia (paracetamol or metamizol) will be introduced, and evaluated effective if a Numeric Pain Rating Scale (NPRS) score is lower than 3 points.

During postoperative care, continuous intraabdominal pressure (IAP) monitoring via a direct intraperitoneal catheter, placed before closure of the abdominal wall, will be performed to eliminate bias caused by the elevation of IAP.

Patients’ clinical progress and secondary endpoints will be monitored by daily SOFA scores, laboratory and physical examinations.

Postoperative hydration and vasopressor therapy will be directed by MAP, ScvO_2_, dCO_2_ and arterial lactate levels. PRBC units will be transfused if decreased hemoglobin (Hb) levels result in tissue oxygenation disorders or become symptomatic (hypotension, dizziness or weakness develop). Fresh frozen plasma will be given if the prothrombin International Normalized Ratio (INR) > 1.5. Platelet suspension units will be given according to the Transfusion Guidelines of the Hungarian National Blood Transfusion Service.

In both groups, patients will be allowed to drink clear fluids immediately after surgery and the use of chewing gum will be encouraged. Prokinetics and an oral liquid diet using a drinking formula will be started on POD 1 and patients will begin active mobilization. The nasogastric tube will be removed on the morning of POD 1.

#### From postoperative day 4 (POD 4 to POD 28, follow-up)

During the follow-up period, secondary endpoints, in-hospital stay, 28-day and in-hospital mortality will also be evaluated.

**Fig. 2:**
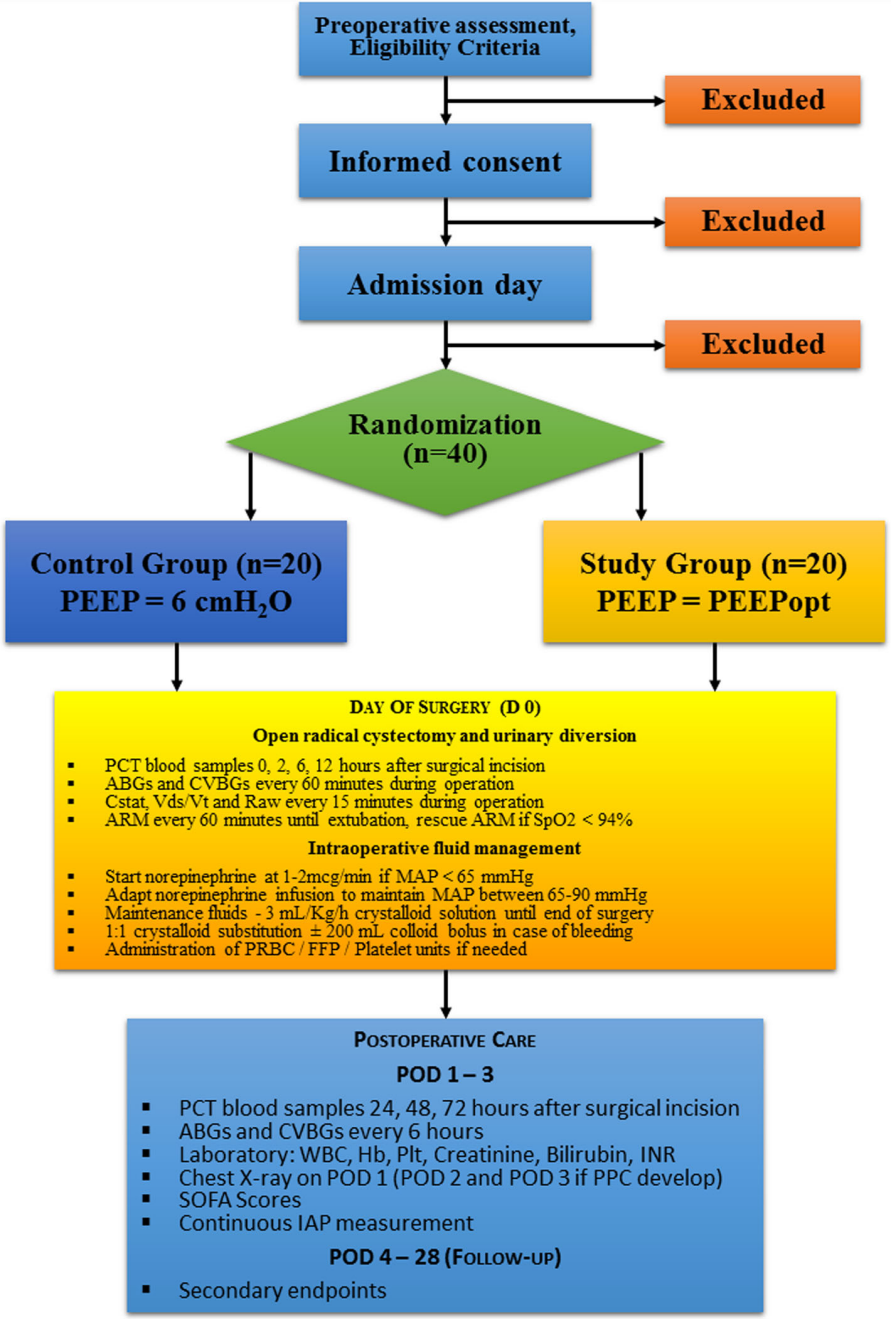
Consolidated Standards of Reporting Trials (CONSORT) flowchart. *PEEP* positive end-expiratory pressure, *PCT* procalcitonin, *ABGs* arterial blood gas sample, *CVBGs* central venous blood gas sample, *Cstat* static pulmonary compliance, *Vds/Vt* dead space fraction, *Raw* airway resistance, *MAP* mean arterial pressure, *ARM* alveolar recruitment maneuver*, PRBC* packed red blood cell, *FFP* fresh frozen plasma, *IAP* intraabdominal pressure

Figure 2 shows the CONSORT flowchart of the trial.

### Study arms and assigned intraoperative interventions

A total number of 40 patients with bladder cancer submitted to general anesthesia and open radical cystectomy and urinary diversion will be enrolled in this study. An equal number of patients will be randomized into the two groups.

Patients randomized into the SG group undergo an alveolar recruitment maneuver using the sustained airway pressure by the CPAP method, applying 30 cmH_2_O PEEP for 30 s followed by a decremental PEEP titration procedure directed by Cstat. During the PEEP titration procedure, PEEP will be decreased from 14 cmH_2_O by 2 cmH_2_O every 4 min, until a final PEEP of 6 cmH_2_O is reached. On each level of PEEP, ABGs will be collected and evaluated. Optimal PEEP is considered as the PEEP value resulting the highest possible Cstat measured by the ventilator. After the PEEP titration procedure, lung-protective mechanical ventilation will be performed using optimal PEEP and low tidal volumes and ARM will be performed every 60 min.

Patients randomized into the CG group will undergo an alveolar recruitment maneuver using the sustained airway pressure by the CPAP method, applying 30 cmH_2_O PEEP for 30 s followed by low-tidal-volume LPV using a PEEP value of 6 cmH_2_O and ARM will be repeated every 60 min.

### Data monitoring

Data monitoring will be performed centrally for quality control purposes by an external, independent physician, who will not be involved in the study. Monitoring will evaluate the progress of the study and verify the accuracy and completeness of the data recording (CRF, source data, Informed Consent Forms and outcome variables).

### Statistics

Data will be analyzed by the research team in collaboration with a medically versed biostatistician after completion of the trial. There will be no interim analysis. Statistical analysis will be conducted on an intention-to-treat basis. IBM SPSS 20.0 statistical software will be used for analysis.

It is expected that the majority of source data will be recorded onto CRF; nonetheless, before starting the data analysis, the mechanism and pattern of missing data will be evaluated and these findings will be used to determine whether they have had an impact on the statistical analysis and results and how they can be managed.

Data distribution will be tested by the Kolmogorov-Smirnov analysis. Normally distributed data will be presented as mean and standard deviation (SD) and skewed data as median (interquartile range). Comparing related samples, the paired and unpaired *t* test will be used for normally distributed data and the Wilcoxon signed rank test and Mann-Whitney *U* test for skewed data. Differences in proportions will be evaluated using the Fisher’s exact test, and risk ratio with associated 95% confidence interval (CI). Analysis of the primary endpoint (PPC) will be carried out by the unpaired Student’s *t* test (95% CI). A two-way, repeated-measures analysis of variance (two-way RM ANOVA) will be used to compare the groups’ serum PCT levels. The relationship between PCT levels and organ dysfunctions will be evaluated using the Pearson correlation. Statistical analysis of SOFA scores, ICU days, in-hospital stay, in-hospital and 28-day mortality data of groups will be implemented by the chi-square test. A *P* value < 0.05 will be considered significant.

### Adverse events and interruption of the trial

Every patient included in the trial will receive daily visits from an intensive care therapist and urologist in charge from POD 1 until leaving the hospital. During ICU stay, and if necessary on the intermediate care unit, all patients will be continuously monitored. The study nurse will be responsible for collecting blood samples and will record relevant required data onto CRF. During the out-of-hospital follow-up period (until POD 28) patients’ progress, particularly deterioration will be checked by daily phone-call visits.

The investigators will monitor the patients for any adverse events (AEs), which are defined as severe or prolonged hypotension (systolic blood pressure < 90 mmHg) and significant cardiac arrhythmias associated with the PEEP titration procedure. AEs will be documented on the CRF and the principal investigator will be informed.

Serious adverse events (SAEs) are defined as severe barotrauma leading to pneumothorax, significant prolongation of hospitalization, persistent or significant disability or incapacity, and severe deterioration (life-threatening state or even death) associated with the PEEP titration procedure. All treatment-related SAEs will be recorded and reported to the Hungarian Scientific and Medical Research Council Ethics Committee and the Local Ethics Committees. If any SAEs occur, the trial will be interrupted and an investigation will be performed.

### Duration of the trial

The annual number of open radical cystectomy and urinary diversion is around 100 in the two study centers. Recruitment of the participants is expected within 18 months. The final data collection and estimated completion date of the trial is March 2018.

## Discussion

This investigator-initiated, pragmatic, interventional, prospective, randomized controlled trial will assess the possible benefits and disadvantages of an individualized lung-protective mechanical ventilation strategy during open radical cystectomy and urinary diversion as indicated mainly by PPC and the inflammatory response.

PPC can develop after major abdominal surgery. Impaired gas exchange may lead to secondary disorders (delayed return of gastrointestinal function, renal dysfunction, cardiac disorders, etc.) resulting in prolonged hospitalization time and increased cost of hospital care [15–17]. The impact of an inappropriate intraoperative mechanical ventilation-caused inflammatory response – both systemic and intrapulmonary –, on these complications is still uncertain.

Surgery induces an inflammatory response that is necessary for postoperative recovery [18–21]. Inappropriate mechanical ventilation can also cause an inflammatory response, which can lead to AEs such as pulmonary complications and distant organ dysfunction. Applying an individualized lung-protective ventilatory strategy during general anesthesia may reduce the degree of inflammation and decrease the incidence of pulmonary and extrapulmonary complications in the postoperative period, thereby contributing to shorter hospitalization time and reduced cost of hospital care [3–5].

Radical cystectomy and urinary diversion is considered major surgery with an operating time lasting for several hours. This gives the potential for inappropriate intraoperative ventilatory management causing further harm by exacerbating the surgery-induced inflammatory response, hence causing more postoperative complications. Titrating PEEP and performing regular ARMs during the anesthesia of these patients certainly has a strong pathophysiological rationale with potential benefits as indicated by recent clinical trials [4–7, 14], but this strategy is also cumbersome, time consuming and, due to the numerous blood gas samplings required, may be costly. Therefore, testing our hypothesis in a clinical study is necessary to answer these questions.

The potential implications of our results can further improve our knowledge on the effects of optimal intraoperative ventilatory strategies and, in the case of positive results, these may not only be applicable to patients with bladder cancer undergoing radical cystectomy and urinary diversion, but presumably to all patients undergoing similar types of major abdominal surgery.

### Trial status

The trial is ongoing.

## Additional file

Additional file 1: SPIRIT 2013 Checklist: recommended items to address in a clinical trial protocol and related documents. (DOCX 52 kb)

## Abbreviations

ABGs: Arterial blood gas samples
ARDS: Acute respiratory distress syndrome
ARM: Alveolar recruitment maneuver
ASA: American Society of Anesthesiologists
BE: Base excess
BMI: Body Mass Index
CG: Control group
CI: Confidence interval
COPD: Chronic obstructive pulmonary disease
CPAP: Continuous positive airway pressure
CRF: Case Report Form
Cstat: Static pulmonary compliance
CT: Computer tomography
CVBGs: Central venous blood gas samples
dCO_2_: Central venous-to-arterial carbon dioxide gap
ECG: Electrocardiogram
EtCO_2_: End-tidal carbon dioxide tension
FiO_2_: Fraction of inspired oxygen
GOLD: Global Initiative for Chronic Obstructive Lung Disease
HR: Heart rate
IAP: Intraabdominal pressure
IBW: Ideal bodyweight
ICU: Intensive care unit
INR: International Normalized Ratio
LMWH: Low-molecular-weight heparin
LPV: Lung-protective ventilation
MAP: Mean arterial pressure
NPRS: Numeric Pain Rating Scale
NRS 2002: Nutritional risk screening
NYHA: New York Heart Association
PaO_2_: Partial pressure of arterial oxygen
PCT: Procalcitonin
PEEP: Positive end-expiratory pressure
POD: Postoperative day
PPC: Postoperative pulmonary complications
PRBC: Packed red blood cells
Raw: Airway resistance
RFRI: Respiratory Failure Risk Index
ScvO_2_: Central venous oxygen saturation
SD: Standard deviation
SG: Study group
SOFA: Sequential Organ Failure Assessment
SPIRIT: Standard Protocol Items: Recommendation for Interventional Trials
SpO_2_: Oxygen saturation
stHCO^3−^: Standard bicarbonate
TV: Tidal volume
Vds/Vt: Dead space fraction
VILI: Ventilator-induced lung injury
